# The Rising Power of Electrochemotherapy in Musculoskeletal Oncology

**DOI:** 10.3390/curroncol33030143

**Published:** 2026-02-28

**Authors:** Nicolas Papalexis, Giuliano Peta, Simone Quarchioni, Laura Campanacci, Alessandro Gasbarrini, Giuseppe Tedesco, Michela Carta, Maddalena Di Carlo, Marco Miceli, Giancarlo Facchini

**Affiliations:** 1 Department of Diagnostic and Interventional Radiology, IRCCS Istituto Ortopedico Rizzoli, 40136 Bologna, Italy; giuliano.peta@ior.it (G.P.); simone.quarchioni@ior.it (S.Q.); michela.carta@ior.it (M.C.); maddalena.dicarlo@ior.it (M.D.C.); marco.miceli@ior.it (M.M.); giancarlo.facchini@ior.it (G.F.); 2 3rd Orthopaedic and Traumatologic Clinic Prevalently Oncologic, IRCCS Istituto Ortopedico Rizzoli, 40136 Bologna, Italy; laura.campanacci@ior.it; 3 Department of Spine Surgery, IRCCS Istituto Ortopedico Rizzoli, 40136 Bologna, Italy; alessandro.gasbarrini@ior.it (A.G.), giuseppe.tedesco@ior.it (G.T.)

**Keywords:** electrochemotherapy, electroporation, electrosclerotherapy, bone neoplasms, soft tissue neoplasms, palliative care, bone metastases, chronic pain, vascular malformation

## Abstract

Electrochemotherapy is a minimally invasive treatment based on the principle of reversible electroporation of target cells in pathologic tissues in order to increase the local effect of chemotherapeutic agents. The mechanism of action relies on temporarily increasing cell permeability to increase the uptake of cytotoxic drugs in the intracellular space. Originally developed for the treatment of cutaneous malignancies, electrochemotherapy has significantly evolved over the past few decades, thanks to advancements in electrode design and image guidance, finding fertile ground in musculoskeletal oncological pathologies, such as bone and soft tissue tumors and different kinds of vascular malformations. Moreover, initial experiences have reported on the treatment of other soft tissue tumors such as desmoid fibromatosis. The aim of this review is to summarize the literature on the role of electrochemotherapy across a variety of musculoskeletal conditions, starting from established oncologic indications, such as metastatic bone or soft tissue tumors, to emerging evidence on primary musculoskeletal pathology, with particular attention paid to the results of the leading studies relating to the efficacy, complications, and recurrence rate.

## Simple Summary

Electrochemotherapy is a minimally invasive technique that enhances the effectiveness of chemotherapy by increasing cell membrane permeability through reversible electroporation. It is now on the rise in the treatment of various lesions, including musculoskeletal tumors, bone and soft tissue malignancies, and vascular malformations. This review summarizes current literature on its applications in musculoskeletal conditions, highlighting evidence on efficacy, safety, and recurrence rates.

## 1. Introduction

Electrochemotherapy (ECT) is a relatively new loco-regional treatment that uses high-voltage electric pulses to induce reversible electroporation and transient increased cell membrane permeability to selectively enhance the uptake of hydrophilic chemotherapeutic drugs, most often Bleomycin, in the target tissue volume [1,2]. The concept of electrochemotherapy originated in the late 1980s, when experimental studies by Okino, Orlowski et al. demonstrated that high-voltage electric pulses markedly enhance the antitumor activity of chemotherapeutic agents such as bleomycin in vivo [3,4].

The term “electrochemotherapy” was subsequently introduced by Mir et al. in the early 1990s to describe this combined modality [5].

This treatment was originally developed for patients with cutaneous and subcutaneous malignancies and, thanks to its effect, has gained increasing attention as a minimally invasive option in the field of interventional oncology [6]. Its mechanism of action relies on a dual effect: the transient permeabilization of the cell membrane to facilitate the intracellular delivery of large molecules of chemotherapeutic agents, while the electric pulses induce vasoconstriction that facilitates local drug concentration and contributes to tumor necrosis through vascular disruption [7,8].

The publication of the ESOPE guidelines in 2006 [9], later updated in 2018 [10], marked a key turning point by standardizing protocols for drug administration, electric pulse delivery, and patient selection [1]. These developments accelerated the adoption of ECT in clinical oncology and laid the groundwork for its expansion beyond superficial diseases.

In recent years, advances in electrode design and the integration of image-guided techniques have allowed for the safe and effective application of ECT to deep-seated tumors. Early feasibility studies demonstrated that long needle electrodes combined with patient-specific planning and navigation systems could achieve adequate tumor coverage while minimizing damage to surrounding tissues [11,12], granting a place for this technique in the musculoskeletal tumor board [13,14,15]. Bone metastases, metastatic primitive bone and soft tissue sarcomas, and desmoid-type fibromatosis have emerged as promising indications, especially for those patients who might have contraindications or limited benefits from surgery or radiation therapy [16,17]. ECT can provide a local antitumor effect while being minimally invasive, making it especially useful in anatomically complex or functionally critical areas such as the spine, the pelvis, or near critical neurovascular structures [18].

ECT does not work just by providing a local cytotoxic action; in fact, some research has shown that it can stimulate systemic antitumor immunity through the release of tumor antigens and damage-associated molecular patterns, leading to dendritic cell activation and a potential abscopal effect. For this reason, combining ECT with immunotherapy may be a future strategy to synergize the effects of the two treatments [19,20]. In addition to drug uptake and immune stimulation, ECT exerts a clinically relevant antivascular effect, characterized by an immediate “vascular lock” (transient vasoconstriction that reduces washout and increases local drug exposure) and a delayed vascular-disrupting action mediated by damage to tumor endothelium, contributing to ischemia and tumor necrosis. This vascular component is considered one of the key mechanisms supporting local control and symptom palliation in selected lesions [21].

Furthermore, recent evidence suggests that ECT may also enhance the effects of radiotherapy, acting as a radiosensitizer by increasing DNA damage and reactive oxygen species generation, thereby offering new potential for combined modality protocols in selected musculoskeletal tumors [22,23].

The aim of this review is to provide a comprehensive overview of the role of electrochemotherapy in musculoskeletal oncology, from well-established applications to emerging indications. We will analyze the available clinical data regarding efficacy, safety, and recurrence rates in bone and soft tissue tumors, and explore the immunological implications of ECT in cancer treatment. By summarizing current evidence, this review aims to clarify the potential of electrochemotherapy as a transformative tool in the multidisciplinary management of musculoskeletal neoplasms.

## 2. Technical Aspects of Electrochemotherapy

Electrochemotherapy (ECT) is a percutaneous locoregional treatment that relies on the synergistic effect of reversible electroporation and chemotherapy. In order to have successful treatment, drug delivery and electrical pulse application must be precisely controlled, and advanced image guidance and electrode design have evolved to deliver a safe and effective antitumor therapy. Although first developed for superficial malignancies, advances in equipment, planning, and electrode engineering have enabled ECT to evolve into a deep-tissue, image-guided intervention with applications ranging from bone metastases to soft tissue sarcomas and vascular malformations (Table 1).

### 2.1. Chemotherapeutic Agents and Administration

The cornerstone of ECT is the use of hydrophilic, poorly membrane-permeant cytotoxic drugs such as bleomycin or cisplatin. Bleomycin is the preferred agent due to its steep increase in cytotoxicity after membrane permeabilization, up to 1000-fold. Drug administration can be intravenous (the most standardized route, typically 8 min before electroporation) or intratumoral, which may be preferred for small, superficial, or vascular lesions to reduce systemic toxicity [10].

In the treatment of low-flow vascular malformations, bleomycin is used at lower concentrations (typically 250–1000 IU/mL), injected intralesionally, and combined with electroporation to enhance endothelial destruction; hence the name bleomycin electrosclerotherapy (BEST) [24,25]. For high-flow vascular malformation, perilesional injection around the nidus was described with very promising results [26].

### 2.2. Electric Pulses and Electroporation Parameters

Short, high-voltage pulses are used to create temporary pores in the cell membrane, a process known as reversible electroporation. The standard parameters, established by the ESOPE guidelines and validated across tumor types, include 8 pulses of 100 μs each and an electric field intensity of 1000–1300 V/cm. In clinical electrochemotherapy, the electric field amplitude is determined by electrode geometry and inter-electrode distance, and is therefore not uniform within the treated tissue. For example, plate electrodes typically operate around 960 V, whereas needle electrodes require lower voltages (≈400–730 V) depending on configuration. The effective electric field decreases with distance from the electrodes, and adequate tumor coverage with sufficiently high local field strength is essential for treatment efficacy. Consequently, treatment planning and electrode selection are tailored to tumor size, location, and depth to ensure appropriate field distribution throughout the target volume [10,13,19,27].

### 2.3. Electrode Design and Geometry

Effective electrochemotherapy requires adequate electric field coverage of the entire tumor volume. The distribution of the electric field depends on electrode geometry, spacing, and placement, which determine whether all tumor regions receive a field strength sufficient to induce reversible electroporation. For this reason, several electrode configurations have been developed for superficial and deep-seated tumors, and treatment success is strongly dependent on appropriate electrode selection and positioning [28,29].

Electrode design plays a central role in ECT efficacy. Two main types are used:

1. Plate electrodes with fixed geometry, suitable for superficial lesions (e.g., cutaneous metastases, superficial soft tissues)
2. Needle electrodes, required for deep, irregular, or large tumors

A major breakthrough in deep tissue ECT came with the introduction of long needle variable geometry electrodes [21]. These allow customizable placement under image guidance to treat large tumors (up to 10 cm) with safe electric field coverage. Needle spacing and insertion depth must be carefully planned to ensure uniform electroporation without exceeding thermal thresholds [19,30,31].

In vascular malformations, needle electrodes are often inserted parallel or tangential to the lesion, with adjustments based on the venous flow pattern and proximity to skin or nerves [27,32]. In high-flow AVMs, some authors have combined arterial catheterization with ECT, enabling selective drug perfusion followed by immediate electroporation for targeted endothelial ablation [33].

### 2.4. Image Guidance and Navigation

Deep-seated tumors and vascular lesions necessitate precise imaging for treatment planning, needle placement, and real-time monitoring. Ultrasound is ideal for superficial or compressible lesions, as it is fast, inexpensive, and offers real-time guidance of the needle.

CT or cone-beam CT are used for bone metastases and soft tissue tumors that are large, deeply located or near neurovascular or critical structures. MRI has its role too, since can be used for volumetric assessment pre- and post-treatment, especially in sarcomas or spinal cases [34]. Moreover, MR guidance is used for cryoablation or biopsies in highly specialized centers [35,36]; however, specific reports on MR-guidance for electrochemotherapy are not yet available.

Image fusion and navigation systems are increasingly being used to simulate electric field distribution and optimize electrode placement, particularly in complex regions such as the pelvis, retroperitoneum, and spine [37].

In addition to conventional image guidance, patient-specific treatment planning tools based on numerical modeling of electric field distribution have been developed to optimize electrode configuration and pulse parameters, particularly for deep-seated tumors. These approaches use imaging-derived anatomical models and finite-element simulations to predict adequate electroporation coverage while minimizing exposure of surrounding critical structures, thereby improving treatment safety and efficacy [38,39,40].

### 2.5. Anesthesia and Patient Preparation

Due to involuntary muscle contractions and potential discomfort from the electric pulses, ECT is typically performed under general anesthesia or deep sedation with muscle relaxation. Local anesthesia may suffice for superficial lesions treated with plate electrodes. For BEST procedures, especially in children or for facial AVMs [26], the choice of anesthesia is dictated by lesion depth, location, and patient cooperation [19,27].

**Table 1:** Procedural workflow of an ECT session.

Phase	Notes
Pre-procedural imaging and planning	CT, MRI, or US for anatomical assessment and electrode planning
Drug administration (IV or intralesional)	Typically bleomycin; timing critical (e.g., 8 min before pulses for IV)
Needle electrodes require imaging guidance for deep targets; plate electrodes are used for superficial lesions and generally do not require imaging for localization.	Needle or plate electrodes, tailored to lesion location and depth
Delivery of electric pulses	Standard ESOPE parameters or adjusted for lesion type/setting
MRI is preferred for local treatment assessment; PET/CT is reserved for selected oncologic indications.	Follow-up imaging depends on indication; PET often used in sarcomas

### 2.6. Safety and Technical Considerations

ECT has demonstrated a favorable safety profile. The most common side effects are skin hyperpigmentation, local swelling, transient pain, and skin ulceration [15]. In BEST, skin necrosis may occur if electrodes are placed too close to the dermis or if the bleomycin dose is excessive [27]. Electroporation must be carefully modulated near critical structures such as nerves, the spinal cord, or major vessels, using lower voltages or narrower pulse widths if needed [37]. In deep-seated soft tissue lesions, extended necrosis and subsequent tissue loss have also been reported and should be taken into account during treatment planning and post-procedural management [41].

Electroporation is contraindicated in patients with uncontrolled seizures or known allergy to bleomycin [27].

## 3. Immunomodulatory and Radiosensitizing Effects of ECT

Early experimental and clinical investigations demonstrated that electrochemotherapy can potentiate radiotherapy and modulate antitumor immune responses through vascular disruption, enhanced DNA damage, and tumor microenvironment changes [42,43,44,45]. Moreover, recent studies have confirmed an immune system modulation and radiosensitization effect of ECT, beyond the direct cytotoxic and vascular-disruptive effects previously described. These findings provide new insight into how ECT can be integrated with systemic therapies and radiotherapy in oncologic care.

### 3.1. Immunomodulatory Effects

ECT may trigger immunogenic cell death, leading to the release of damage-associated molecular patterns such as ATP, HMGB1, and calreticulin. These molecules act as immunological danger signals that activate dendritic cells and promote antigen presentation, potentially leading to a systemic antitumor immune response [2].

Preclinical and clinical evidence of ECT’s immunostimulatory effects is summarized in a recent comprehensive review [23]. In murine models, ECT stimulates dendritic cell activity and T-cell recruitment. Clinical studies in melanoma patients have demonstrated increased CD8+ T-cell infiltration and decreased regulatory T-cells in treated lesions. In some cases the abscopal effect has been observed, supporting the hypothesis that ECT can act as an in situ vaccine. These properties suggest that ECT may be a valuable companion to immune checkpoint inhibitors or cytokine-based therapies [23,46].

Moreover, growing preclinical and early clinical evidence supports the rationale for combining electrochemotherapy with immunotherapy. Preclinical data have demonstrated that ECT-induced immunogenic cell death may act as an in situ vaccination, enhancing dendritic cell activation and T-cell priming, thereby potentiating the efficacy of immune checkpoint inhibitors [47]. More recent translational and clinical observations further suggest that the addition of immunotherapy to ECT may improve both local tumor control and systemic antitumor responses, particularly in advanced melanoma and other solid tumors [2].

### 3.2. Radiosensitizing Effects

In addition to its immune-stimulating properties, ECT seems to enhance the effects of radiotherapy. A systematic review [14,42] concluded that ECT may increase tumor radiosensitivity via multiple mechanisms. The enhanced drug uptake following electroporation leads to increased DNA damage, including single- and double-strand breaks, and the generation of reactive oxygen species, which augment the cytotoxic effects of ionizing radiation.

Preclinical studies have shown that a single ECT session prior to irradiation can improve tumor response, and similar findings have been reported in early-phase clinical observations. These synergistic effects offer an opportunity to combine ECT with single-dose or fractionated radiotherapy protocols, especially in radio-resistant tumors or when minimizing the total radiation dose is desired [14,42].

## 4. Electrochemotherapy in Bone Metastases

Bone is the third most common landing site for metastatic lesions, with the spine being the most frequently affected skeletal site. The longer life expectancy of patients with advanced skeletal disease [48,49] imposes a strategic management of bone metastases, creating a therapeutic challenge for those patients who are not eligible for or would not benefit from surgery, or for patients with lesions in close proximity to vital structures, which could make other minimally invasive treatments such as cryoablation or embolization too risky [50]. On the other hand, the frequent presence of symptoms such as pain, bone instability, or neurologic impairment creates the need for a minimally invasive treatment capable of palliating pain in fragile and often pluri-metastatic patients [51]. Electrochemotherapy (ECT), by leveraging the synergistic effects of electric pulses and cytotoxic drugs, has recently emerged as a minimally invasive approach for local control of metastatic bone disease [52]. The most relevant articles related to ECT in bone metastases are summarized in Table 2. 

### 4.1. Spinal Metastases and Spinal Epidural Invasion

The first reported application of ECT in the spine dates back to a 2015 case report by Gasbarrini et al. [53], who treated a melanoma spinal metastasis at L5 with intraoperative ECT combined with decompressive surgery. The patient experienced significant improvement in pain and quality of life, with no severe complications during the 48-month follow-up period.

Subsequent studies have explored the percutaneous, image-guided application of ECT in the spine. In 2019, Cornelis et al. [55] reported the feasibility and efficacy of cone beam CT-guided ECT in two patients with posterior vertebral metastases and epidural extension. Pain relief and local tumor control were achieved in both cases without neurological complications.

**Figure 1:**
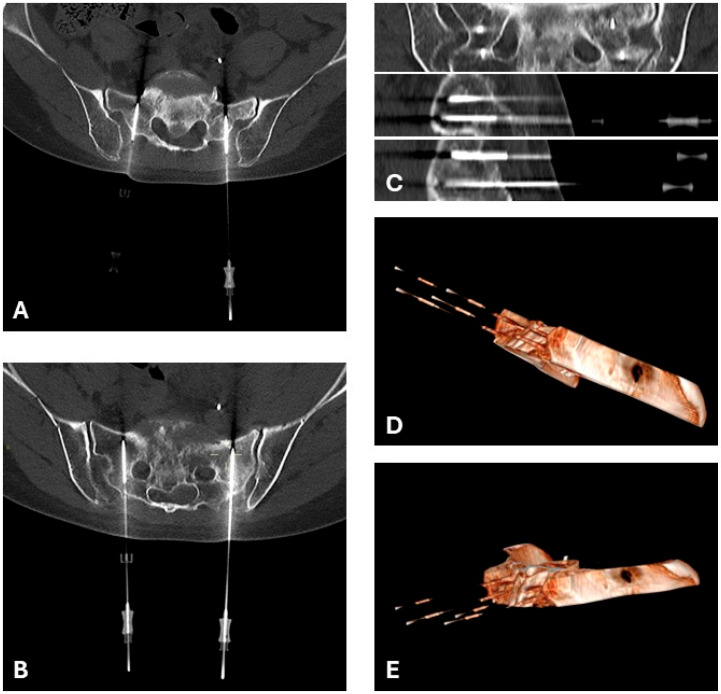
A 51-year-old female, affected by colon adenocarcinoma, presented at our Institute with an osteolytic lesion of the sacrum. The patient was treated with electrochemotherapy. CT scans (**A**,**B**) and 3D (**D**,**E**) reconstructions show the percutaneous positioning of the four 18 VGD needle electrodes (30 mm active tip) following a parallel geometry (**C**), allowing the entire lesion to be treated. Bleomycin was injected intravenously, and electroporation was performed. No adverse events were recorded after the procedure. The patient reported clinical improvement at the 3-month follow-up.

**Figure 2:**
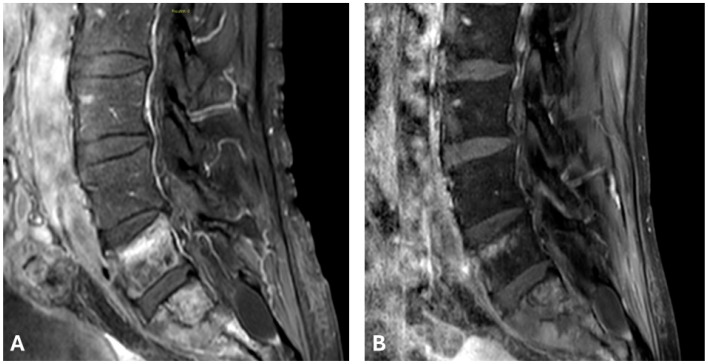
Contrast-enhanced T1-weighted fat-saturated MRI sequences at baseline show contrast uptake in lesions at the levels of L5, S1 and S2, as well as in the extracompartmental component of S1 (**A**). The same sequences, at the 3-month follow-up, display a reduction in vertebral and extracompartmental contrast uptake (**B**).

Recent systematic efforts have focused on spinal metastases, where ECT is increasingly adopted in cases unresponsive to radiotherapy or in patients who are not surgical candidates (Figure 1 and Figure 2). Deschamps et al. [34] analyzed 40 patients with metastatic epidural spinal cord compression who had previously been treated with radiation. ECT was able to achieve local control and provide pain relief in 80% of patients. Neurological symptoms improved in over half the cohort, and 77% of patients showed a radiologic response by 1-month MRI. However, they reported temporary acute radicular pain in 25% of treated patients, prolonged radicular hypoesthesia in 10%, and paraplegia in 7.5% [34].

Angelini et al. [18] presented a case series combining ECT with decompressive laminectomy and spinal fixation. Their protocol involved transpedicular placement of electrodes during open surgery and demonstrated the potential to integrate ECT into complex surgical strategies, especially in cases with mechanical instability or neural compression [18].

Lastly, Deschamps et al. [37] reviewed the existing literature on ECT for metastatic epidural spine compression, finding that pain relief and local tumor control were achieved in most cases. At three months, complete or partial response was observed in over 65% of patients, with minimal procedure-related complications [37]. 

### 4.2. Extra-Spinal Bone Metastases

Among the first clinical trials to explore electrochemotherapy in bone was the phase II study by Bianchi et al. [54] published in 2016. The trial included 29 patients with painful bone metastases primarily involving the pelvis (18 cases), humerus (6), and femur (5). All patients had previously received local treatments, such as radiotherapy or embolization, without sufficient symptom control. ECT was performed under fluoroscopic or CT guidance using needle electrodes and intravenous bleomycin. The results were encouraging: 84% of patients reported a ≥50% reduction in pain, and 73% experienced improvement in daily functioning and sleep. Follow-up imaging showed stable disease in the majority of cases, and there were no significant procedural complications. The study demonstrated for the first time the technical feasibility and safety of ECT in deep and weight-bearing bones, without compromising structural integrity [54].

Building on the first experience, the most comprehensive study to date on the application of electrochemotherapy in bone metastases is the multicenter prospective registry published in 2021 by Campanacci et al. [31], which included a cohort of 102 patients with painful bone metastases who were not suitable for surgery or radiotherapy, or had already failed those treatments. Lesions were most frequently located in the pelvis, femur, and ribs, with a smaller number involving the spine. The trial aimed to establish standardized operating procedures for the percutaneous application of ECT in bone and to assess its safety, efficacy, and clinical results on pain, function, and quality of life. In their study, 24 patients (23.5%) underwent planned intramedullary nail fixation during the same operative session as ECT. The mean follow-up was 5.9 ± 5.1 months (range: 1.5–52 months). Pain response and tumor control were evaluated using both RECIST and PERCIST criteria. Their results were very promising, with objective responses achieved in approximately 30–40% of patients, while most others achieved disease stabilization [31].

The combination of ECT and intramedullary fixation was specifically investigated in a recent prospective study by Cevolani et al. [57], which included a cohort of 32 patients with femoral, humeral, or tibial metastases. Thirteen patients had pathological fractures and 19 had impending ones. The study showed a significant decrease in pain in 79% of patients. Bone recovery, as assessed radiologically, was observed in 50% of evaluable cases, and fracture healing was achieved in 73% of fractured lesions. Importantly, no severe systemic complications were observed, and only one intraoperative fracture occurred. These findings support the synergistic effect of combining ECT and internal fixation, especially in cases requiring mechanical stabilization alongside local tumor control [57].

An important contribution to the evidence base was made by Bocchi et al. [15], who conducted the first systematic review specifically dedicated to ECT for bone metastases, encompassing data from eight studies and a total of 246 patients treated for 250 lesions. Their analysis confirmed the safety and clinical utility of ECT in a wide range of skeletal locations, particularly in the appendicular skeleton (54.8%) and pelvis (26.7%), but also included spinal sites (20.8%). Breast, kidney, and lung cancers were the most frequent primary tumors. The review reported a significant reduction in pain, with mean VAS scores dropping from 6.9 pre-treatment to 2.7 post-treatment. Tumor control was observed in the majority of patients: RECIST-based outcomes revealed complete and partial responses in 12% and 26.1% of cases, respectively, with stable disease in over half of the cohort (55.5%). Complications were infrequent (9.7%), and no systemic adverse events were reported [15].

## 5. Electrochemotherapy in Soft Tissue Tumors

Soft tissue tumors include a heterogeneous group of benign and malignant neoplasms that may arise in virtually any anatomical compartment, often presenting as deep-seated or infiltrative masses [58]. Their management is frequently complex, especially in cases where lesions are non-resectable, located near critical structures, or recurrent after surgery or radiotherapy. Despite the availability of systemic therapies and targeted agents, locoregional control remains a major challenge [59]. 

Electrochemotherapy (ECT) has progressively established itself as a valuable tool in the locoregional treatment of soft tissue tumors, offering a non-thermal, tissue-preserving alternative particularly suited for anatomically complex, previously treated, or non-resectable lesions [1]. In addition to human clinical studies, electrochemotherapy has also been investigated in the veterinary field for spontaneously occurring soft tissue sarcomas. In a cohort of dogs with incompletely excised STSs treated with systemic bleomycin combined with local cisplatin electroporation, the approach was well tolerated and associated with prolonged disease-free survival, supporting the biological efficacy and translational relevance of ECT in soft tissue malignancies [60].

### 5.1. ECT in Soft Tissue Sarcomas

One of the first prospective trials assessing ECT in advanced or metastatic soft tissue sarcomas was the phase II study by Campana et al. [61], which demonstrated an objective response rate exceeding 90%, including both complete and partial responses. Most patients had previously undergone surgery or radiation, and many were treated for local recurrence. The study confirmed the tolerability and efficacy of standard ECT using intravenous bleomycin and plate or needle electrodes depending on lesion depth [61].

Following the first experience, Simioni et al. [30] treated 30 patients with non-resectable soft tissue tumors, mostly metastatic soft tissue sarcomas and metastatic melanomas, with a median tumor diameter of 4.7 cm, using variable geometry needle electrodes under CT or ultrasound guidance. At one-month follow-up, a complete response (CR) was observed in 63%, while partial response (PR) occurred in 27%, resulting in a 90% overall response rate and a median reduction of SUV in PET-CT of 86%. Clinical benefit was significant, with improvements in pain, mobility, and quality of life. Only one case of grade 3 toxicity was reported (local skin ulceration), and no systemic adverse events occurred [30].

A follow-up study by Ottlakan et al. [17] reinforced these findings, showing that ECT using variable geometry electrodes was effective in treating large, inoperable sarcomas in anatomically constrained regions. The study reported 7 cases, with 5 showing partial response, 1 with stable disease and 1 with progressive disease. The study supports the feasibility and efficacy of ECT as a limb- and function-preserving option in high-risk sarcoma patients who otherwise had no curative alternatives [17].

### 5.2. ECT in Desmoid Fibromatosis

The term “desmoid fibromatosis” (DF), also referred to as “desmoid tumor” or “aggressive fibromatosis,” is an uncommon and locally invasive monoclonal fibroblastic proliferation characterized by a variable and often unpredictable clinical course [62]. It represents less than 3% of soft tissue tumors; its incidence is estimated at 3 to 5 per million, with a peak occurrence between the ages of 30 and 40, and a higher prevalence in women. The majority of DF are sporadic and located extra-abdominally in the trunk or extremities [63,64]. Given the unpredictable progression of DF, characterized by the potential for tumors to either proliferate or remain stable, or even regress partially or completely, updated guidelines advocate for an initial strategy of watchful waiting [65]. 

Intervention is necessary if there is evidence of growth or symptomatic tumors. Current protocols recommend choosing between local therapies, such as percutaneous cryoablation, which is one of the most recent and effective options available [66], and systemic treatments, which mainly consist of chemotherapy and tyrosine kinase inhibitors, including Pazopanib [67]. Electrochemotherapy has also been reported as a feasible treatment option for aggressive fibromatosis (desmoid tumors). In a case report, ECT with intravenous bleomycin resulted in significant tumor reduction and durable symptom relief, with tumor size decreasing from 7.1 × 2.2 cm to 2.9 × 1.7 cm at one-year follow-up and marked pain improvement. This suggests that ECT may represent a minimally invasive local therapy for selected patients with unresectable or symptomatic desmoid tumors [16].

## 6. Electrochemotherapy and Electrosclerotherapy in Vascular Malformations 

Vascular malformations are congenital anomalies of blood or lymphatic vessels that may involve the skin, soft tissues, or deep anatomical compartments. Depending on flow dynamics, they are classified as low-flow, venous or lymphatic, or high-flow, mostly arteriovenous malformations (AVMs), each posing different diagnostic and therapeutic challenges [68,69]. While some lesions remain stable or asymptomatic, others may cause pain, discomfort, swelling, bleeding, esthetic concerns, or functional impairment, particularly when located near nerves, joints, or in the head and neck. Standard treatments include sclerotherapy and embolization [70,71], which may not always be effective, and surgery, which carries a high risk of morbidity, especially in high-flow or infiltrative lesions [24].

In recent years ECT has been adapted specifically for vascular malformations, acquiring the name of electrosclerotherapy [25]. This emerging treatment is effective and minimally invasive, with very few side effects. In most cases, bleomycin is injected directly into the vascular malformation for low-flow lesions or into the surrounding soft tissues for high-flow vascular malformations. Electric pulses are then delivered using insulated plate fixed electrodes (most commonly) or variable geometry electrodes [25,27]. The main studies on electrosclerotherapy for VMs are summarized in Table 3.

### 6.1. Low Flow Vascular Malformations

In 2021, the earliest clinical evidence supporting the use of bleomycin electrosclerotherapy in vascular malformations was published by Wohlgemuth et al. [25]. The study involved 17 patients (mean age: 27.4 years) with therapy-resistant venous malformations, all of whom had failed prior interventions such as sclerotherapy, laser ablation, or surgery. The procedure was performed using intralesional injection of bleomycin at a concentration of 1 IU/mL, with the total dose adapted to lesion size (typically not exceeding 15 IU per session). Following the injection, electric pulses were applied through percutaneously inserted needle electrodes, spaced based on lesion geometry. Imaging guidance, primarily ultrasound, was used for electrode positioning and drug distribution monitoring. Electroporation was performed shortly (within 1–2 min) after bleomycin injection to maximize endothelial uptake. The technique was named BEST, and this name was subsequently used to describe it.

The results were promising, with an average lesion volume reduction of 86%, while clinical symptom resolution was achieved in 47% of patients, with partial improvement in most others. No systemic side effects or long-term complications were reported, and the procedure was well tolerated under local or general anesthesia, depending on lesion location and patient age [25].

These findings were further supported by a larger cohort study published by Schmidt et al. [32], which examined BEST in both adults and children with slow-flow vascular malformations. Across 49 procedures in 36 patients, the authors documented clinical improvement in over 80% of cases, with significant volume reduction on MRI and no permanent side effects. The treatment was found to be particularly beneficial in deep or anatomically complex malformations, where conventional surgery or sclerotherapy would be high-risk.

The growing use of BEST has led to the development of standardized technical protocols, including the Current Operating Procedure published by Tobian Muir [27], which describes step-by-step procedural guidelines, reporting bleomycin dosage, electrode geometry, pulse delivery timing, and post-treatment monitoring.

In addition to its effectiveness as monotherapy, BEST has also been evaluated in combination with polidocanol foam sclerotherapy [69]. When comparing BEST + foam with foam alone, the BEST group demonstrated significantly higher response rates (73% vs. 52%) and improved symptom control, especially in large or multi-compartmental lesions. No increase in toxicity was observed.

### 6.2. High Flow Vascular Malformations

More recently, interest has expanded into the treatment of high-flow malformations. These lesions are notoriously difficult to manage due to their complex shunting patterns and high risk of recurrence after embolization or surgery. In 2022, Krt et al. [33] described a new hybrid approach combining superselective arterial catheterization with ECT, allowing for targeted delivery of bleomycin into high-flow AVMs of the head and neck, followed by intranodal electroporation. Under angiographic guidance, microcatheters were navigated into the arterial feeders of the AVM, and bleomycin was infused directly into the nidus. Immediately afterward, needle electrodes were percutaneously inserted around the lesion under ultrasound or fluoroscopic guidance, and electric pulses were delivered according to ESOPE parameters. This method was shown to reduce flow and alleviate symptoms, even in previously embolized lesions [33].

Latini et al. [72] also reported on two patients with large head and neck AVMs treated with fractionated bleomycin and BEST. Both cases showed a marked reduction in Doppler vascularity and clinical symptoms, with excellent tolerability. These findings have encouraged the cautious expansion of ECT into the realm of high-flow vascular malformations, albeit in highly selected patients and at specialized centers [72].

A modified version of BEST, referred to as MEST (modified electrosclerotherapy), was introduced by Colletti et al. [26] for the treatment of high-flow vascular malformations. This technique builds upon the principles of BEST but incorporates specific technical adjustments aimed at improving efficacy and safety in high-flow lesions. Bleomycin was administered intralesionally or perilesionally with multiple injections to ensure adequate diffusion throughout the AVM nidus. The pulses were then applied in a fractionated, stepwise fashion, allowing for controlled coverage of the lesion with reduced electrical burden per session. The authors reported favorable results in terms of flow reduction, symptom control, and cosmetic improvement, with no significant adverse events. Although limited to pilot cases, the MEST approach offers a promising refinement of electrosclerotherapy, particularly for AVMs located in high-risk or aesthetically sensitive areas, where standard electroporation parameters might be too aggressive [26].

## 7. Electrochemotherapy for Aggressive Vertebral Hemangioma

Vertebral hemangiomas are the most common primary spinal bone lesion, affecting approximately 10–12% of the population [73]. Usually asymptomatic, hemangiomas are accidental findings that do not require treatment [74]. They are referred to as locally aggressive or “aggressive hemangiomas” if they spread past the vertebral body to the spinal canal or neural foramina [75], eventually developing symptoms, including back pain and neurologic impairments [74]. Radiotherapy, isolated decompressive surgery, vertebroplasty, vascular embolization, or a combination of these treatments are available for symptomatic or locally aggressive lesions [76,77].

Recent evidence suggests that electrochemotherapy can serve as a salvage treatment when standard options fail. In a case series, Tedesco et al. [78] described the application of ECT with intravenous bleomycin in two patients with recurrent, symptomatic vertebral hemangiomas previously treated with surgery, radiotherapy, and embolization. The procedure employed transpedicular or paravertebral insertion of insulated electrodes under image guidance, followed by standard electroporation protocols. One patient showed a reduction in lesion size and spinal canal compression after two ECT sessions, while the other maintained lesion stability after a single session, with improved pain control. Notably, both treatments were completed without complications, confirming the feasibility and safety of ECT in spinal vascular tumors, particularly where thermal ablation or reoperation pose high risks. This study opens the door to using ECT in selected benign but aggressive vascular lesions of the spine, traditionally considered off-limits for loco-regional therapies [78].

## 8. Discussion

Over the last decade, electrochemotherapy has emerged as a valuable tool in the multidisciplinary treatment of a wide variety of musculoskeletal tumors and in the complex field of vascular malformations. Originally developed for cutaneous malignancies, the technique has progressively been adapted for deeper, more complex lesions thanks to technological improvements in electrode design, image guidance, and treatment planning. This review summarizes the vast landscape of the many applications of ECT in musculoskeletal oncology, primarily in bone and soft tissue metastases, primary soft tissue tumors, and both low- and high-flow vascular malformations.

In the complex multidisciplinary management of patients with bone metastases, often having limited options due to comorbidities, prior treatments, or anatomical constraints, ECT has demonstrated consistent pain relief and local tumor control. The early work by Bianchi et al. [54] provided proof of concept for deep skeletal lesions, later expanded upon by Campanacci et al. [31] with a standardized, multicenter protocol. The combination of ECT with intramedullary fixation offers a synergistic approach that maintains mechanical stability while achieving oncologic control [31,57]. Compared with other percutaneous treatment options for bone metastases, electrochemotherapy offers a non-thermal mechanism of action that may be advantageous in anatomically constrained locations and in proximity to critical neural or vascular structures. Unlike thermal ablation techniques, ECT does not rely on heat diffusion, potentially reducing the risk of collateral damage in sensitive areas such as the spine or pelvis. While cementoplasty primarily addresses mechanical stabilization and pain relief, ECT may contribute to local tumor control and can be integrated into combined strategies tailored to both oncologic and biomechanical goals. Although direct comparative data remain limited, these characteristics support a complementary role of ECT within the spectrum of minimally invasive treatments for metastatic bone disease [31,37].

In cases of spinal involvement, conventional ablation techniques might be limited by the need for preserving neurologic function and avoiding thermal injury; therefore, ECT might be a good alternative in complex cases and has shown encouraging results in both percutaneous and intraoperative settings. Even in extreme cases such as spinal epidural extension [18,34], ECT may still be feasible, expanding the therapeutic arsenal for managing metastatic spinal cord compression.

The role of ECT in soft tissue tumors is also growing, especially for lesions deemed inoperable due to size, depth, or proximity to critical structures. Recent studies have demonstrated that variable geometry electrodes and image-guided planning enable the safe treatment of large sarcomas and aggressive fibromatosis with high response rates and minimal morbidity [17,30,61]. The only available case report underscores the potential role of ECT in deeply located desmoid-type fibromatosis of the limbs [16]. While the evidence is very limited, the broad application of the technique to other soft tissue tumors will most likely catalyze new evidence specifically for desmoids.

The application of ECT in the vast territory of vascular malformations, often still treated surgically with high morbidity, represents a significant conceptual leap. Originally applied to low-flow venous malformations, where traditional sclerotherapy had failed [25], bleomycin electrosclerotherapy now offers high-volume reduction and symptom control in deep or surgically inaccessible lesions [2,23]. The development of standardized protocols, such as the BEST Current Operating Procedure [27], reflects growing clinical acceptance [27]. Furthermore, early exploratory work in high-flow vascular malformations points toward an expanding frontier where arterial catheterization and tailored electroporation strategies may open new therapeutic pathways for the most challenging vascular anomalies [26,72]. Additionally, recent prospective data suggest that ECT may improve or maintain quality of life in patients with smaller or previously irradiated lesions, reinforcing its value in palliative oncology settings [79]. 

Despite the encouraging results across multiple indications, several limitations should be acknowledged. Most studies to date are small, single-arm trials or retrospective series, and randomized controlled trials are still lacking. Moreover, the long-term durability of tumor control, optimal integration with systemic therapies, and immunomodulatory synergy remain areas of active investigation. 

## 9. Conclusions

In conclusion, ECT has evolved into a versatile and well-tolerated technique with expanding applications in musculoskeletal oncology and vascular anomalies. It offers a minimally invasive, anatomy-preserving alternative for malignancies in patients with few remaining options, or as a standalone curative treatment for patients with vascular malformations or selected benign soft tissue tumors. Future directions should focus on protocol optimization, broader clinical validation, and translational research into its immunologic and radiosensitizing potential. As evidence grows, ECT may well become an essential component in the multidisciplinary management of complex loco-regional disease.

## Figures and Tables

**Table 2:** Main studies on electrochemotherapy for bone metastases.

Year	First Author	Patients	Study Type	Site	Technique	Key Highlights
2015	Gasbarrini [53]	1	Case Report	Spinal	Intra-op ECT + decompression	First spinal case; pain/QoL improvement; no complications
2016	Bianchi [54]	29	Phase II Trial	Extra-spinal	ECT + IV bleomycin under fluoroscopy/CT	84% ≥ 50% pain relief; safe in deep bones
2019	Cornelis [55]	2	Feasibility Study	Spinal	CBCT-guided percutaneous ECT	Feasibility in epidural involvement; no neuro injury
2021	Campanacci [31]	102	Prospective Multicenter	Mixed	ECT ± intramedullary fixation	30–40% objective response; nail fixation in 23.5% cases
2022	Campanacci [56]	38	Prospective Monocentric Registry	Extra-spinal	ECT ± intramedullary fixation	Objective response: 29% (RECIST), 36% (PERCIST); 68% reported pain reduction;
2023	Cevolani [57]	32	Prospective Study	Extra-spinal	ECT + intramedullary fixation	79% pain relief; 73% fracture healing
2023	Deschamps [34]	40	Retrospective Study	Spinal	ECT in MESCC	80% pain relief; 77% radiologic response; some neuro deficits
2023	Bocchi [15]	246	Systematic Review	Mixed	Pooled ECT in bone metastases	VAS ↓ from 6.9 to 2.7; CR/PR 38%; low complication rate
2024	Angelini [18]	3	Case Series	Spinal	Transpedicular needle electrodes during surgery + IV bleomycin.	Pain relief and neurologic improvement; stable disease on imaging

**Table 3:** Main studies reporting on electrochemotherapy/electrosclerotherapy for VMs.

Year	First Author	Patients	Study Type	Malformation Type	Technique	Key Highlights
2021	Wohlgemuth [25]	17	Case Series	Slow-flow VM	Intralesional bleomycin + ECT (BEST)	First clinical evidence; 86% lesion volume reduction; well tolerated
2022	Krt [33]	1	Feasibility Study	High-flow AVM	Superselective intra-arterial bleomycin + perilesional ECT	Effective flow reduction, complete remission
2023	Liu [69]	152	Comparative Study	Slow-flow VM	Polidocanol foam ± BEST	Both BPF and ECP are effective treatments for MVs, with BPF being a safer option
2024	Schmidt [32]	233	Retrospective Study	Slow-flow VM	Intralesional/intravenous bleomycin + ECT (BEST)	BEST improved function and aesthetics, reducing VAS in 41.6% and eliminating it in 17.7%.
2024	Muir [27]	NA	Guidelines/COP	Slow-flow VM	Standardized BEST protocol	Defines bleomycin dosage, electrode type, pulse timing
2025	Latini [72]	2	Case Report	High-flow AVM	Fractionated BEST	Reduced vascularity and symptoms; high tolerability
2025	Colletti [26]	10	Pilot Study	High-flow AVM	Modified ECT protocol (MEST) with fractionated pulses and reduced voltage	Prototype method with symptom improvement and cosmetic benefit

## Data Availability

No new data were created or analyzed in this study.

## Abbreviations

The following abbreviations are used in this manuscript:

ECT	Electrochemotherapy
AVM	Arteriovenous Malformation
BEST	Bleomycin Electrosclerotherapy

## References

[1] Probst U., Fuhrmann I., Beyer L., Wiggermann P. (2018). Electrochemotherapy as a New Modality in Interventional Oncology: A Review. Technol. Cancer Res. Treat..

[2] Hadzialjevic B., Omerzel M., Trotovsek B., Cemazar M., Jesenko T., Sersa G., Djokic M. (2023). Electrochemotherapy Combined with Immunotherapy—A Promising Potential in the Treatment of Cancer. Front. Immunol..

[3] Orlowski S., Belehradek J., Paoletti C., Mir L. (1988). Transient electropermeabilization of cells in culture- increase of the cyto-toxicity of anticancer drugs. Biochem. Pharmacol..

[4] Okino M., Mohri H. (1987). Effects of a high-voltage electrical impulse and an anticancer drug on invivo growing tumors. Jpn. J. Cancer Res..

[5] Mir L.M., Orlowski S., Belehradek J., Paoletti C. (1991). Electrochemotherapy potentiation of antitumour effect of bleomycin by local electric pulses. Eur. J. Cancer.

[6] Spugnini E.P., Citro G., D’Avino A., Baldi A. (2008). Potential role of electrochemotherapy for the treatment of soft tissue sarcoma: First insights from preclinical studies in animals. Int. J. Biochem. Cell Biol..

[7] Jarm T., Cemazar M., Miklavcic D., Sersa G. (2010). Antivascular Effects of Electrochemotherapy: Implications in Treatment of Bleeding Metastases. Expert Rev. Anticancer. Ther..

[8] Ramirez L.H., Orlowski S., An D., Bindoula G., Dzodic R., Ardouin P., Bognel C., Belehradek J., Munck J.N., Mir L.M. (1998). Electrochemotherapy on Liver Tumours in Rabbits. Br. J. Cancer.

[9] Mir L.M., Gehl J., Sersa G., Collins C.G., Garbay J.-R., Billard V., Geertsen P.F., Rudolf Z., O’sUllivan G.C., Marty M. (2006). Standard Operating Procedures of the Electrochemotherapy: Instructions for the Use of Bleomycin or Cisplatin Administered Either Systemically or Locally and Electric Pulses Delivered by the Cliniporator^TM^ by Means of Invasive or Non-Invasive Electrodes. Eur. J. Cancer Suppl..

[10] Gehl J., Sersa G., Matthiessen L.W., Muir T., Soden D., Occhini A., Quaglino P., Curatolo P., Campana L.G., Kunte C. (2018). Updated Standard Operating Procedures for Electrochemotherapy of Cutaneous Tumours and Skin Metastases. Acta Oncol..

[11] Grošelj A., Kos B., Čemažar M., Urbančič J., Kragelj G., Bošnjak M., Veberič B., Strojan P., Miklavčič D., Serša G. (2015). Coupling treatment planning with navigation system: A new technological approach in treatment of head and neck tumors by electrochemotherapy. Biomed. Eng. Online.

[12] Miklavčič D., Snoj M., Županič A., Kos B., Čemažar M., Kropivnik M., Bračko M., Pečnik T., Gadžijev E., Serša G. (2010). Towards treatment planning and treatment of deep-seated solid tumors by electrochemotherapy. Biomed. Eng. Online.

[13] Miklavčič D., Mali B., Kos B., Heller R., Serša G. (2014). Electrochemotherapy: From the drawing board into medical practice. Biomed. Eng. Online.

[14] Papalexis N., Parmeggiani A., Peta G., Spinnato P., Miceli M., Facchini G. (2022). Minimally Invasive Interventional Procedures for Metastatic Bone Disease: A Comprehensive Review. Curr. Oncol..

[15] Bocchi M.B., Meschini C., Pietramala S., Perna A., Oliva M.S., Matrangolo M.R., Ziranu A., Maccauro G., Vitiello R. (2023). Electrochemotherapy in the Treatment of Bone Metastases: A Systematic Review. J. Clin. Med..

[16] Vitfell-Rasmussen J., Sandvik R.M., Dahlstrøm K., Al-Farra G., Krarup-Hansen A., Gehl J. (2018). Tumor Reduction and Symptom Relief after Electrochemotherapy in a Patient with Aggressive Fibromatosis—A Case Report. Acta Oncol..

[17] Ottlakan A., Lazar G., Hideghety K., Koszo R.L., Deak B., Nagy A., Besenyi Z., Bottyan K., Vass G.Z., Olah J. (2022). Clinical Considerations of Bleomycin Based Electrochemotherapy with Variable Electrode Geometry Electrodes for Inoperable, Deep-Seated Soft Tissue Sarcomas. Bioelectrochemistry.

[18] Angelini A., D’Amico A., Paolilli S., Signori R., Baldin G., Di Rubbo G., Denaro L., Ruggieri P. (2024). Electrochemotherapy in Spine Metastases: A Case Series Focused on Technical Aspects, Surgical Strategies and Results. Diagnostics.

[19] Campana L.G., Edhemovic I., Soden D., Perrone A.M., Scarpa M., Campanacci L., Cemazar M., Valpione S., Miklavčič D., Mocellin S. (2019). Electrochemotherapy—Emerging Applications Technical Advances, New Indications, Combined Approaches, and Multi-Institutional Collaboration. Eur. J. Surg. Oncol..

[20] Barca I., Ferragina F., Kallaverja E., Cristofaro M.G. (2023). Synergy of Electrochemotherapy and Immunotherapy in the Treatment of Skin Squamous Cell Carcinoma of the Head and Neck. Oral Maxillofac. Surg. Cases.

[21] Campana L.G., Miklavčič D., Bertino G., Marconato R., Valpione S., Imarisio I., Dieci M.V., Granziera E., Cemazar M., Alaibac M. (2019). Electrochemotherapy of Superficial Tumors—Current Status. Semin. Oncol..

[22] Rezaee Z., Yadollahpour A., Rashidi S., Kunwar P.S. (2017). Radiosensitizing Effect of Electrochemotherapy: A Systematic Review of Protocols and Efficiency. Curr. Drug Targets.

[23] Ferioli M., Perrone A.M., Buwenge M., Arcelli A., Vadala’ M., Fionda B., Malato M.C., De Iaco P., Zamagni C., Cammelli S. (2023). Combination of Electrochemotherapy with Radiotherapy: A Comprehensive, Systematic, PRISMA-Compliant Review of Efficacy and Potential Radiosensitizing Effects in Tumor Control. Curr. Oncol..

[24] Muir T., Bertino G., Groselj A., Ratnam L., Kis E., Odili J., McCafferty I., Wohlgemuth W.A., Cemazar M., Krt A. (2023). Bleomycin Electrosclerotherapy (BEST) for the Treatment of Vascular Malformations. An International Network for Sharing Practices on Electrochemotherapy (InspECT) Study Group Report. Radiol. Oncol..

[25] Wohlgemuth W.A., Müller-Wille R., Meyer L., Wildgruber M., Guntau M., von der Heydt S., Pech M., Zanasi A., Flöther L., Brill R. (2021). Bleomycin Electrosclerotherapy in Therapy-Resistant Venous Malformations of the Body. J. Vasc. Surg. Venous Lymphat. Disord..

[26] Colletti G., Rozell-Shannon L., Nocini R. (2025). MEST: Modified Electrosclerotherapy to Treat AVM (Extracranial Arterio-Venous Malformations). Better than BEST. J. Craniomaxillofac. Surg..

[27] Muir T., Wohlgemuth W.A., Cemazar M., Bertino G., Groselj A., Ratnam L.A., McCafferty I., Wildgruber M., Gebauer B., de Terlizzi F. (2024). Current Operating Procedure (COP) for Bleomycin ElectroScleroTherapy (BEST) of Low-Flow Vascular Malformations. Radiol. Oncol..

[28] Miklavčič D., Serša G., Brecelj E., Gehl J., Soden D., Bianchi G., Ruggieri P., Rossi C.R., Campana L.G., Jarm T. (2012). Electrochemotherapy: Technological advancements for efficient electroporation-based treatment of internal tumors. Med. Biol. Eng. Comput..

[29] Geboers B., Scheffer H.J., Graybill P.M., Ruarus A.H., Nieuwenhuizen S., Puijk R.S., van den Tol P.M., Davalos R.V., Rubinsky B., de Gruijl T.D. (2020). High-voltage electrical pulses in oncology: Irreversible electroporation, electrochemotherapy, gene electrotransfer, electrofusion, and electroimmunotherapy. Radiology.

[30] Simioni A., Valpione S., Granziera E., Rossi C.R., Cavallin F., Spina R., Sieni E., Aliberti C., Stramare R., Campana L.G. (2020). Ablation of Soft Tissue Tumours by Long Needle Variable Electrode-Geometry Electrochemotherapy: Final Report from a Single-Arm, Single-Centre Phase-2 Study. Sci. Rep..

[31] Campanacci L., Bianchi G., Cevolani L., Errani C., Ciani G., Facchini G., Spinnato P., Tognù A., Massari L., Cornelis F.H. (2021). Operating Procedures for Electrochemotherapy in Bone Metastases: Results from a Multicenter Prospective Study on 102 Patients. Eur. J. Surg. Oncol..

[32] Schmidt V.F., Cangir Ö., Meyer L., Goldann C., Hengst S., Brill R., von der Heydt S., Waner M., Puhr-Westerheide D., Öcal O. (2024). Outcome of Bleomycin Electrosclerotherapy of Slow-Flow Malformations in Adults and Children. Eur. Radiol..

[33] Krt A., Cemazar M., Lovric D., Sersa G., Jamsek C., Groselj A. (2022). Combining Superselective Catheterization and Electrochemotherapy: A New Technological Approach to the Treatment of High-Flow Head and Neck Vascular Malformations. Front. Oncol..

[34] Deschamps F., Tselikas L., Yevich S., Bonnet B., Roux C., Kobe A., Besse B., Berthelot K., Gaudin A., Mir L.M. (2023). Electrochemotherapy in Radiotherapy-Resistant Epidural Spinal Cord Compression in Metastatic Cancer Patients. Eur. J. Cancer.

[35] Cazzato R.L., Garnon J., Ramamurthy N., Tsoumakidou G., Caudrelier J., Thenint M.-A., Rao P., Koch G., Gangi A. (2016). Percutaneous MR-Guided Cryoablation of Morton’s Neuroma: Rationale and Technical Details after the First 20 Patients. Cardiovasc. Radiol..

[36] Autrusseau P.-A., Cazzato R.L., De Marini P., Dalili D., Koch G., Boatta E., Auloge P., Garnon J., Gangi A. (2020). Percutaneous MR-Guided Cryoablation of Low-Flow Vascular Malformation: Technical Feasibility, Safety and Clinical Efficacy. Cardiovasc. Radiol..

[37] Deschamps F., Tselikas L., Cazzato R.L., Facchini G., Granata V., Bonnet B., D’Alessio V., Fusco R., Zanasi A., de Terlizzi F. (2025). Electrochemotherapy in Metastatic Epidural Spinal Cord Compression: A Review and Technical Update. Br. J. Radiol..

[38] Cindrič H., Miklavčič D., Cornelis F.H., Kos B. (2022). Optimization of transpedicular electrode insertion for electroporation-based treatments of vertebral tumors. Cancers.

[39] Cindrič H., Kos B., Tedesco G., Cadossi M., Gasbarrini A., Miklavčič D. (2018). Electrochemotherapy of spinal metastases using transpedicular approach—A numerical feasibility study. Technol. Cancer Res. Treat..

[40] Perera-Bel E., Aycock K.N., Salameh Z.S., Gómez-Barea M., Davalos R.V., Ivorra A., González Ballester M.A. (2023). PIRET—A platform for treatment planning in electroporation-based therapies. IEEE Trans. Biomed. Eng..

[41] Lenzi R., Muscatello L., Saibene A.M., Felisati G., Pipolo C. (2017). The controversial role of electrochemotherapy in head and neck cancer: A systematic review of the literature. Eur. Arch. Otorhinolaryngol..

[42] Serša G., Kranjc S., Čemažar M. (2000). Improvement of combined modality therapy with cisplatin and radiation using electroporation of tumors. Int. J. Radiat. Oncol. Biol. Phys..

[43] Serša G., Čemažar M., Rudolf Z., Fras A.P. (1999). Adenocarcinoma skin metastases treated by electrochemotherapy with cisplatin combined with radiation. Radiol. Oncol..

[44] Serša G., Teissié J., Čemažar M., Signori E., Kamenšek U., Marshall G., Miklavčič D. (2015). Electrochemotherapy of tumors as in situ vaccination boosted by immunogene electrotransfer. Cancer Immunol. Immunother..

[45] Calvet C.Y., Famin D., Andre F.M., Mir L.M. (2014). Electrochemotherapy with bleomycin induces hallmarks of immunogenic cell death in murine colon cancer cells. Oncoimmunology.

[46] Campana L.G., Peric B., Mascherini M., Spina R., Kunte C., Kis E., Rozsa P., Quaglino P., Jones R.P., Clover A.J.P. (2021). Combination of pembrolizumab with electrochemotherapy in cutaneous metastases from melanoma: A comparative retrospective study from the InspECT and Slovenian Cancer Registry. Cancers.

[47] Calvet C.Y., Mir L.M. (2016). The promising alliance of anti-cancer electrochemotherapy with immunotherapy. Cancer Metastasis Rev..

[48] Errani C., Bazzocchi A., Spinnato P., Facchini G., Campanacci L., Rossi G., Mavrogenis A.F. (2019). What’s New in Management of Bone Metastases?. Eur. J. Orthop. Surg. Traumatol..

[49] Rogers D.L., Raad M., Rivera J.A., Wedin R., Laitinen M., Sørensen M.S., Petersen M.M., Hilton T., Morris C.D., Levin A.S. (2024). Life Expectancy after Treatment of Metastatic Bone Disease: An International Trend Analysis. J. Am. Acad. Orthop. Surg..

[50] Cazzato R.L., Auloge P., Dalili D., De Marini P., Di Marco A., Garnon J., Gangi A. (2019). Percutaneous Image-Guided Cryoablation of Osteoblastoma. AJR Am. J. Roentgenol..

[51] Hong S., Youk T., Lee S.J., Kim K.M., Vajdic C.M. (2020). Bone Metastasis and Skeletal-Related Events in Patients with Solid Cancer: A Korean Nationwide Health Insurance Database Study. PLoS ONE.

[52] Mali B., Jarm T., Snoj M., Sersa G., Miklavcic D. (2013). Antitumor Effectiveness of Electrochemotherapy: A Systematic Review and Meta-Analysis. Eur. J. Surg. Oncol..

[53] Gasbarrini A., Campos W.K., Campanacci L., Boriani S. (2015). Electrochemotherapy to Metastatic Spinal Melanoma: A Novel Treatment of Spinal Metastasis?. Spine.

[54] Bianchi G., Campanacci L., Ronchetti M., Donati D. (2016). Electrochemotherapy in the Treatment of Bone Metastases: A Phase II Trial. World J. Surg..

[55] Cornelis F.H., Ben Ammar M., Nouri-Neuville M., Matton L., Benderra M.A., Gligorov J., Fallet V., Mir L.M. (2019). Percutaneous Image-Guided Electrochemotherapy of Spine Metastases: Initial Experience. Cardiovasc. Interv. Radiol..

[56] Campanacci L., Cevolani L., De Terlizzi F., Saenz L., Alì N., Bianchi G., Donati D.M. (2022). Electrochemotherapy Is Effective in the Treatment of Bone Metastases. Curr. Oncol..

[57] Cevolani L., Campanacci L., Staals E.L., Dozza B., Bianchi G., De Terlizzi F., Donati D.M. (2023). Is the Association of Electrochemotherapy and Bone Fixation Rational in Patients with Bone Metastasis?. J. Surg. Oncol..

[58] World Health Organization (2013). WHO Classification of Tumours of Soft Tissue and Bone.

[59] Kurup A.N., Callstrom M.R. (2013). Ablation of Musculoskeletal Metastases: Pain Palliation, Fracture Risk Reduction, and Oligometastatic Disease. Tech. Vasc. Interv. Radiol..

[60] Spugnini E.P., Vincenzi B., Amadio B., Baldi A. (2019). Adjuvant electrochemotherapy with bleomycin and cisplatin combination for canine soft tissue sarcomas: A study of 30 cases. Open Vet. J..

[61] Campana L.G., Bianchi G., Mocellin S., Valpione S., Campanacci L., Brunello A., Donati D., Sieni E., Rossi C.R. (2014). Electrochemotherapy Treatment of Locally Advanced and Metastatic Soft Tissue Sarcomas: Results of a Non-Comparative Phase II Study. World J. Surg..

[62] Desmoid Tumor Working Group (2020). The Management of Desmoid Tumours: A Joint Global Consensus-Based Guideline Approach for Adult and Paediatric Patients. Eur. J. Cancer.

[63] Mikhael R., Smith M., Tzanis D., Watson S., Miah A.B., Bonvalot S. (2022). Desmoid Tumors: Who, When and How to Treat?. Curr. Opin. Oncol..

[64] Kasper B., Baldini E.H., Bonvalot S., Callegaro D., Cardona K., Colombo C., Corradini N., Crago A.M., Dei Tos A.P., Dileo P. (2024). Current Management of Desmoid Tumors: A Review. JAMA Oncol..

[65] Kasper B., Baumgarten C., Garcia J., Bonvalot S., Haas R., Haller F., Hohenberger P., Penel N., Messiou C., van der Graaf W.T. (2017). An Update on the Management of Sporadic Desmoid-Type Fibromatosis: A European Consensus Initiative between Sarcoma PAtients EuroNet (SPAEN) and European Organization for Research and Treatment of Cancer (EORTC)/Soft Tissue and Bone Sarcoma Group (STBSG). Ann. Oncol..

[66] Garnon J., Cazzato R.L., Autrusseau P.-A., Koch G., Weiss J., Gantzer J., Kurtz J.-E., Gangi A. (2024). Desmoid Fibromatosis: IR (sometimes) to the Rescue for an Atypical Disease. Br. J. Radiol..

[67] Testa S., Bui N.Q., Charville G.W., Avedian R.S., Steffner R., Ghanouni P., Mohler D.G., Ganjoo K.N. (2022). Management of Patients with Newly Diagnosed Desmoid Tumors in a First-Line Setting. Cancers.

[68] Wassef M., Blei F., Adams D., Alomari A., Baselga E., Berenstein A., Burrows P., Frieden I.J., Garzon M.C., Lopez-Gutierrez J.-C. (2015). Vascular Anomalies Classification: Recommendations from the International Society for the Study of Vascular Anomalies. Pediatrics.

[69] Liu J.-W., Ni B., Gao X.-X., He B., Nie Q.-Q., Fan X.-Q., Ye Z.-D., Wen J.-Y., Liu P. (2024). Comparison of Bleomycin Polidocanol Foam vs. Electrochemotherapy Combined with Polidocanol Foam for Treatment of Venous Malformations. J. Vasc. Surg. Venous Lymphat. Disord..

[70] Papalexis N., Peta G., Carta M., Quarchioni S., Di Carlo M., Miceli M., Facchini G. (2024). How Arterial Embolization Is Transforming Treatment of Oncologic and Degenerative Musculoskeletal Disease. Curr. Oncol..

[71] Gowda P.C., Schaar D.A., Gong A.J., Garg T., Khalil A., Weinstein R., Morefield W.F., Bailey C., Weiss C.R. (2025). Clinical and Imaging Outcomes of Doxycycline Exchange Sclerotherapy for Lymphatic Malformations. J. Vasc. Interv. Radiol..

[72] Latini L., Bracco S., Cioni S., Leonini S., Cascino F., Gennaro P. (2025). Innovative Use of Bleomycin Electrosclerotherapy (BEST) for High-Flow Arteriovenous Malformations in the Head District: Preliminary Results of Two Cases. J. Clin. Med..

[73] Slon V., Stein D., Cohen H., Sella-Tunis T., May H., Hershkovitz I. (2015). Vertebral Hemangiomas: Their Demographical Characteristics, Location along the Spine and Position within the Vertebral Body. Eur. Spine J..

[74] Wang B., Meng N., Zhuang H., Han S., Yang S., Jiang L., Wei F., Liu X., Liu Z. (2018). The Role of Radiotherapy and Surgery in the Management of Aggressive Vertebral Hemangioma: A Retrospective Study of 20 Patients. Med. Sci. Monit..

[75] Acosta F.L., Sanai N., Chi J.H., Dowd C.F., Chin C., Tihan T., Chou D., Weinstein P.R., Ames C.P. (2008). Comprehensive Management of Symptomatic and Aggressive Vertebral Hemangiomas. Neurosurg. Clin. N. Am..

[76] Girardo M., Zenga F., Bruno L.L., Rava A., Massè A., Maule M., Fusini F. (2019). Treatment of Aggressive Vertebral Hemangiomas with Poly Vinyl Alcohol (PVA) Microparticles Embolization, PMMA, and Short Segment Stabilization: Preliminary Results with at Least 5 Years of Follow-Up. World Neurosurg..

[77] Vasudeva V.S., Chi J.H., Groff M.W. (2016). Surgical Treatment of Aggressive Vertebral Hemangiomas. Neurosurg. Focus.

[78] Tedesco G., Noli L.E., Griffoni C., Ghermandi R., Facchini G., Peta G., Papalexis N., Asunis E., Pasini S., Gasbarrini A. (2024). Electrochemotherapy in Aggressive Hemangioma of the Spine: A Case Series and Narrative Literature Review. J. Clin. Med..

[79] Rózsa P., Rárosi F., Ócsai H., Baltás E., Oláh J., Kemény L., Gyulai R., Kis E.G. (2025). Quality of Life Changes after Electrochemotherapy: A Prospective Single-Center Analysis. Sci. Rep..

